# Web-Based Physical Activity Intervention for COPD: Differences Between Rural and Urban Veterans

**DOI:** 10.1093/geroni/igaa057.1605

**Published:** 2020-12-16

**Authors:** Stephanie Robinson, Stephanie Shimada, Caroline Richardson, Marilyn Moy

**Affiliations:** 1 Edith Nourse Rogers Memorial Veterans Hospital, Bedford, Massachusetts, United States; 2 University of Michigan, Ypsilanti, Michigan, United States; 3 West Roxburry VAMC, West Roxbury, Massachusetts, United States

## Abstract

Physical activity is recommended for all patients with chronic obstructive pulmonary disease (COPD), a disease prevalent among older adults. Rural Veterans often lack access to in-person services to support behavior change. Technology can be used to overcome these barriers. This secondary analysis of a randomized controlled trial compared engagement (weekly website-logons) and efficacy (step-count change) measures of a 4-month technology-based physical activity intervention between rural and urban Veterans with COPD. Rural-urban commuting codes (RUCA) were used to classify participants (N=239; mean age=66.7±8.84 years) as ‘rural’ or ‘urban’. Participants were randomized 2:1 to a pedometer and website with iterative, individualized step-count feedback and goals, motivational and informational messages, and an online community (intervention group, n=155, 45.8% rural) or to receive a pedometer-alone (waitlist-control group, n=84, 44% rural). General linear modeling estimated the adjusted effect of rurality on intervention response. There was no significant rural-urban difference in weekly website-logons (3.33 vs. 3.17; p=.364). There was a significant interaction between rurality and group on step-count change (p=.045). Among rural participants, both intervention and controls increased their step counts (567.32 vs. 732.30; p=.769). Among urban participants, the intervention group demonstrated a significantly higher change in step-counts compared to controls (1052.67 vs. -313.57; p=.008). Results suggest that rural participants increased their step-counts similarly whether they received a pedometer alone or used the intervention, whereas only urban participants who used the intervention increased their step counts. It is possible that simply receiving the pedometer alone was sufficient for behavior change in the rural participants.

